# Metabolomic profiling, antioxidant activity, and skin cell viability of citrus peel flavonoids extracted via ultrasonic-assisted aqueous two-phase system

**DOI:** 10.1371/journal.pone.0336325

**Published:** 2025-12-12

**Authors:** Shuiqing Lin, Jinmei Hu, Hui He, Yaying Yu, Zhengguo Zhou, Lin Zhou

**Affiliations:** 1 Guangdong Provincial Key Laboratory of Advanced Drug Delivery, Guangdong Provincial Engineering Center of Topical Precise Drug Delivery System, School of Life Sciences and Biopharmaceutics, Guangdong Pharmaceutical University, Guangzhou, China; 2 School of Food Health, College of Guangdong Polytechnic of Environmental Protection Engineering, Foshan, China; 3 Guangzhou Kaihong Flavour & Fragrance Co., Ltd, Guangzhou, China; SRM University AP, INDIA

## Abstract

Citrus peels, often considered agricultural waste, are rich in flavonoids with potent antioxidant properties. This study utilized ultrasonic-assisted aqueous two-phase extraction (UA-ATPE) to obtain flavonoid-rich upper phase extracts (UPEs) from the peels of four citrus varieties sourced from different cultivars and regions. Targeted metabolomics was used to analyze the flavonoid compositions in the UPEs, revealing distinct metabolic profiles among the varieties. The antioxidant activities of the UPEs were evaluated through hydroxyl, superoxide, and ABTS radical scavenging assays, while their effects on skin cell viability were assessed using HaCaT and BJ cells. Multivariate statistical analyses, including principal component analysis (PCA) and hierarchical clustering analysis (HCA), identified six key differential metabolites (naringenin, p-coumaric acid, luteolin, butin, vitexin/isovitexin, and ferulic acid) that effectively distinguished the citrus varieties. Among the UPEs, Guangxi mandarin orange exhibited the highest total flavonoid content and the most potent superoxide anion and ABTS radical scavenging activities. However, Meizhou shatian pomelo demonstrated the strongest overall antioxidant capacity, as indicated by the lowest IC_50_ values for the antioxidant potential composite (APC). Cell viability assays confirmed that none of the UPEs exhibited cytotoxicity at concentrations of 0.06--1.00% (v/v). These findings highlight significant varietal differences in flavonoid content and antioxidant activity, providing a scientific basis for the utilization of citrus peels in functional cosmetics and other industries.

## 1. Introduction

Citrus species (Citrus spp.), members of the Rutaceae family, are among the most widely cultivated and consumed fruits globally, with significant production in countries such as China, Brazil, India, Mexico, the United States, and Spain [1]. Key citrus varieties, including oranges, grapefruits, mandarins, and lemons, are not only valued for their nutritional content but also serve as major industrial crops [2]. Their distinct bioactive profiles influence flavor, nutritional quality, and consumer preferences, thereby shaping market demand [3]. Among citrus fruits, oranges dominate global production, accounting for approximately 54.84% of total output, followed by mandarins (24.70%), lemons/limes (20.44%), and grapefruits (6.47%) [4]. In China, several region-specific citrus cultivars have become industrially important due to their high yield. For instance, Guangxi province accounts for approximately 30% of the national citrus yield, with over 466,600 hectares under cultivation and a total output value exceeding 100 billion RMB, positioning it as the leading mandarin-producing region (available from Produce Report). Meizhou (Guangdong province) produces around 20% of the nation’s pomelos, covering 43,333 hectares (available from China Daily). Jiangxi’s Gannan region (notably Xinfeng and Anyuan counties) yields over 210,000 tons of navel oranges annually [5], while Anyue county in Sichuan supplies 80% of China’s lemon production [6].

Global citrus production has surged in recent years, reaching 158.5 million tons in 2021−2022 [3]. However, this growth has also led to the generation of approximately 10 million metric tons of citrus processing waste annually, with citrus peels constituting 40%−50% of this waste by weight [7]. While citrus peels are often regarded as waste, they are, rich in various bioactive compounds, including pectin, essential oils, fiber, flavonoid-containing phenolics, phenolic acid, and ascorbic acid [8]. Flavonoids, one of the main bioactive components in citrus, can be categorized into polymethoxylated flavones (PMFs, e.g., tangeretin, nobiletin), flavones (e.g., apigenin, luteolin, and diosmetin), flavanones (e.g., naringin, hesperidin, and naringenin), and flavonols (e.g., quercetin and rutin) [9]. PMFs exist almost exclusively in the citrus genus, particularly in the peel of citrus fruits [10]. As rare naturally occurring bioactive compounds, PMFs not only possess unique structural characteristics but also exhibit diverse biological activities, including antioxidant, anti-inflammatory, anticancer, neuroprotective, and lipid-lowering effects [11]. In addition, these flavonoid compounds hold significant economic value. For instance, in 2022, the market prices in Japan for hesperidin, naringin, and neohesperidin were approximately $192, $487, and $142,599 per 100 grams, respectively [12]. Additionally, citrus flavonoids have demonstrated a range of biological activities, including antioxidant, anti-aging, anti-inflammatory, anticancer, antiviral, and neuroprotective effects [13]. Owing to their multiple health-promoting properties, citrus peels have found broad applications in both food and cosmetic industries. In the food industry, they are commonly used as natural preservatives and colorants, owing to their rich content of bioactive compounds such as flavonoids and essential oils [14]. In addition, citrus peels are widely recognized as safe and effective natural antioxidants, capable of scavenging free radicals, inhibiting lipid peroxidation, and reducing oxidative damage. These antioxidant activities help maintain cellular structural and functional integrity [15]. As a result, citrus peel extracts are increasingly incorporated into cosmetic formulations aimed at delaying skin aging and enhancing skin protection.

Flavonoids in citrus fruits exhibit tissue and variety specificity in their distribution, which consequently influences their differences in biological activities. For example, hesperidin is the most abundant flavanone glycoside in citrus peel, characteristic of oranges [16], and is known for its strong free radical scavenging and metal ion reduction properties [17]. In contrast, the main metabolite in pomelo peel is naringin, which effectively protects the skin from UVB-induced keratinocyte apoptosis and damage by inhibiting the production of reactive oxygen species (ROS) and the overexpression of cyclooxygenase-2 (COX-2) [18]. Citrus flavonoids can lighten skin by modulating the enzymes and signaling pathways involved in melanin synthesis. Their antioxidant properties also scavenge free radicals, reducing oxidative damage to skin cells and slowing skin aging [19]. Due to climate and environmental differences, there may be significant variations in the types and chemical compositions of flavonoids in citrus peels from different sources. Thus, to elucidate the composition and content of flavonoids in four common citrus UPEs, targeted metabolomics based on UPLC-MS and multivariate statistical techniques were employed to analyze the flavonoid profiles of different citrus species.

Metabolomics involves the qualitative and quantitative analysis of low molecular weight molecules with a relative molecular mass below 1000 Da [20]. Metabolomics is generally divided into targeted metabolomics and untargeted metabolomics based on research goals. Targeted metabolomics employs standards and isotopic labels to achieve precise qualitative and quantitative analysis of specific metabolites [21]. In contrast, untargeted metabolomics provides a broader analysis of metabolites, albeit with relatively lower precision in qualitative accuracy. Due to the diversity and complexity of citrus peel constituents, achieving comprehensive analysis and absolute quantification simultaneously is challenging. In this study, we selected major flavonoids as target metabolites and conducted targeted metabolic analysis of various citrus peel flavonoids using UPLC-MS.

Although the extraction and isolation of flavonoids from plants are well-established, there are still disadvantages such as poor selectivity, environmental pollution, and high industrial application costs [22]. In contrast, the dual-phase extraction technique is widely used due to its advantages of being environmentally friendly, simple to operate, clear phase separation, and high extraction efficiency [23]. In addition, solvent polarity plays a crucial role in influencing flavonoids solubility, which may have a significant impact on the extraction yield and their activity [24]. Typically, methanol is the most effective solvent for extracting phenolic acids and catechin; ethanol preferentially recovers flavonoids and their glycosides, catechol, and tannins; whereas acetone affords the highest yields of proanthocyanidins and tannins [25]. Although methanol and acetone deliver high flavonoid recoveries, their industrial deployment is discouraged regarding cost, toxicity, and safety problems [26]. To meet sustainability criteria, aqueous ethanol has become the default solvent for industrial flavonoid extraction. This approach not only reduces environmental impact but also lowers industrial costs, making it a greener and more viable option for the recovery of bioactive compounds from citrus peel waste.

However, the extraction efficiency and purity largely depend on the extraction agent system, including the nature and composition of solvents, leading to different extraction options or modes [27]. In the ethanol and potassium hydrogen phosphate extraction system, ethanol, as an organic solvent, can dissolve various flavonoids, while potassium hydrogen phosphate, as a recognized safe salt solution, can also regulate ion strength and pH value, facilitating the separation of different types of flavonoids [28]. This combination adapts to the diversity of flavonoids, improves distribution behavior, and protects the environment. In addition, ultrasound-assisted extraction is often combined with other extraction processes due to its high extraction efficiency and low industrial application costs, aiming to increase the extraction rate of substances and obtain more active compounds [29]. For example, Garcia-Castello et al. [30] extracted flavonoid compounds from grapefruit waste using conventional solid-liquid extraction and ultrasound-assisted extraction. Londono-Londono et al. [31] obtained flavonoid fractions from South American cultivars of lime, orange and tangerine peels with high yield (40.25 ± 12.09 mg of flavonoid fraction/g peel) using an optimized aqueous ultrasound-assisted extraction method. Therefore, to maximize the utilization of active substances in citrus peel, it is feasible to combine ultrasound-assisted technology with dual-phase extraction technology for the simultaneous extraction of flavonoid compounds and pectin from the peel.

This study investigates the correlation between the flavonoid profiles extracted from diverse citrus peels using ultrasonic-assisted aqueous two-phase extraction (UA-ATPE) and their respective antioxidant properties and cell viability. To elucidate the varietal and regional influences on flavonoid profiles, evaluate their potential for valorization as agricultural by-products. Thus, based on a comprehensive consideration of species diversity, regional distribution characteristics, and metabolite differences, we selected four representative citrus cultivars—Guangxi mandarin orange, Meizhou Shatian pomelo, Sichuan lemon, and Jiangxi navel orange—as the research objects. UA-ATPE was employed to prepare flavonoid-rich phases of the upper phases extract. Subsequently, LC-MS was used for targeted metabolomics analysis of fourty flavonoid compounds. The in vitro antioxidant capacity was evaluated by measuring the scavenging abilities against hydroxyl (·OH) radicals, superoxide anion (O_2_^·–^) radicals, and ABTS radicals. Furthermore, the cell viability and safety of HaCaT and BJ cells was assessed to evaluate the possibility of UPE’s application in cosmetics, providing a new pathway for natural and safe cosmetic raw materials. This study innovatively integrates UA-ATPE for simultaneous recovery of flavonoids and pectin, while bridging the lab-to-industry gap through metabolomic profiling and cytotoxicity validation.

## 2. Materials and methods

### 2.1. Chemicals and instrument

Four fresh citrus fruit cultivars, including mandarin orange, Shatian pomelo, lemon, and navel orange (Table 1), were selected as the raw materials for extraction. Ethanol, hydrochloric acid, oxalic acid were purchased from Guangzhou Chemical Reagent Factory (Guangzhou, China). Sodium nitrite, aluminum nitrate solid were purchased from Shanghai Aladdin Biochemical Technology Co., Ltd. (Shanghai, China). Potassium dihydrogen phosphate was purchased from Tianjin Chemical Plant Co., Ltd. (Tianjin, China). Salicylic acid, ferrous sulfate, ascorbic acid, tris(hydroxymethyl)aminomethane, pyrogallol were purchased from Shanghai Macklin Biochemical Co., Ltd. (Shanghai, China). Rutin was purchased from Shanghai Xushuo Biological Technology Co., Ltd. (Shanghai, China). Sodium hydroxide was purchased from Tianjin Fuchen Chemical Reagent Factory (Tianjin, China). Hydrogen peroxide was purchased from Jiangxi Caoshanhu Disinfection Products Co., Ltd. (Jiangxi, China). ABTS was purchased from Biyuntian Biotechnology Co., Ltd. (Shanghai, China).

**Table 1:** Species information of the four citrus.

Cultuvars	Mandarin orange	Shatian pomelo	Lemon	Navel orange
Collect Location	Yulin, Guangxi (22.6°N,110.1°E)	Meizhou, Guangdong (24.3°N,116.1E)	Anyue, Sichuan (30.1°N,105.3°E)	Ganzhou, Jiangxi (25.8°N,114.9°E)
Ecological type	karst terrain	humid subtropical	foggy low-light	red soil hilly areas
Length (cm)	5.90 ± 2.18 b	15.20 ± 2.95 a	6.20 ± 1.93 b	7.30 ± 2.11 b
Width (cm)	4.20 ± 1.97 b	12.30 ± 2.57 a	4.9 ± 1.88 b	7.00 ± 1.90 b
Moisture content (%)	69.58 ± 0.00 c	77.39 ± 0.00 b	85.24 ± 0.03 a	73.13 ± 0.00 c
Skin color	bright orange	light yellow	yellow	orange-red

**Values followed by different letters within the same row are significantly different** (*p* < 0.05).

HaCaT and BJ cell lines were obtained from Guangdong Marubi Biotechnology Co., Ltd. (Guangzhou, China). High-glucose Dulbecco’s Modified Eagle Medium (DMEM), penicillin/streptomycin, trypsin were purchased from Gibco (Grand Island, NY, USA). Fetal bovine serum (FBS) was purchased from Hangzhou Sijiqing Bioengineering Materials Co., Ltd. (Hangzhou, China).

The following equipment was used for the experiment: Multi-function shredder, Yongkang Sufeng Industry and Trade Co., Ltd ((Zhejiang, China);HC-3026R high-speed refrigerated centrifuge, Anhui Zhongke Zhongjia Scientific Instrument Co., Ltd. (Hefei, China); ELX800 microplate reader, Biotek (Phoenix, AZ, USA); Agilent 1290 Infinity LC ultra-high-performance liquid chromatography, Agilent Corporation (California, USA); 5500 QTRAP mass spectrometer, SCIEX, (Shanghai, China); Constant Temperature Water Bath, Suzhou Shuangyuan Biotechnology Co., Ltd. (Suzhou, China); UV-1800 UV-Visible Spectrophotometer, Shimadzu Corporation, (Tokyo, Japan); Ultrasonic Cleaning Machine, Ningbo Xinzhi Biotechnology Co., Ltd. (Zhejiang, China).

### 2.2. Sample preparation

Fresh mandarin oranges, shatian pomelos, lemons, and navel oranges were selected and thoroughly washed with clean water. A small incision was made at the bottom of each fruit to separate the peel. The peels were then cut into small pieces and crushed into fine granules using a blender.

### 2.3. Moisture content

A certain amount of fresh citrus peel granules (4.0 g) was added to a small beaker of 50 mL size. Samples were weighed every 2 h during oven drying at 105 °C until the weight difference between two consecutive measurements was less than 0.01 g, indicating a constant weight.

### 2.4. Ethanol/dipotassium hydrogen phosphate aqueous two-phase system

Based on the preliminary research of our research group, we selected the following methods and parameters for experimentation. A certain amount of fresh citrus peel granules (4.0 g) was added to a conical flask, 50 mL 75%(v/v) ethanol solution, and 50 mL of 60% (w/v) dipotassium hydrogen phosphate solvent was added and extracted through an ultrasonic bath for 30 min at 70°C temperature (80 W, 40 Hz). Pour the mixture into a separatory funnel, let it stand at room temperature for 2 h, then separate the layers and record the volumes of the upper and lower phase solutions.

Collect the extract from the lower phase and filter it. The resulting pomace was washed three times with 50 mL of distilled water to remove any salts. Filter and clean the washed pomace through cheesecloth. Add the filtered pomace to 20 mL of hot water at 80°C in a water bath and maintain at 80°C for 30 min. Filter the mixture while hot. Add 1.5 times the volume of 95% ethanol solution to the filtrate to precipitate the pectin. Filter again to obtain the pectin. Dry the pectin at 50°C until a constant mass is achieved. (Pectin extraction by acid treatment, with the exception of not undergoing the washing steps of UA-ATPE, everything else is as stated above.)


Pectinextractionyield(%)=Weightofpectin÷Weightoffreshcitruspeel×100 (1)

### 2.5. Determination of the main active ingredient content in the UPEs

#### 2.5.1. Total flavonoid quantification.

The total flavonoid content (TFC) was determined using the sodium nitrite-aluminum nitrate-sodium hydroxide color development method [32], with slight modifications. Briefly, 1.0 mL of extract was combined with 0.15 mL of 5% (w/v) sodium nitrite (NaNO_2_) solution and vortexed. After 6 min, 0.15 mL of 10% (w/v) aluminum chloride (AlCl_3_) was mixed, and the solution was allowed to stand for 6 min before the addition of 2.0 mL of 4% (w/v) sodium hydroxide (NaOH) solution. Then, the volume of the mixture was completed to 5.0 mL with 75% (v/v) ethanol, mixed thoroughly and incubated at 25°C for 15 min. The solution absorbance was measured at 510 nm using ELX800 microplate reader. Rutin was used as the standard, the standard curve (Y = 8.0538X + 0.0008, R^2 ^= 0.996) was prepared to determine the total flavonoid content of the UPEs. The flavonoid extraction rate was calculated according to the following formula.


Flavonoidextractionyield(%)=[(C×V×N)÷M]×100 (2)

Where C represents the flavonoid content in the extraction solution (mg/mL); V denotes the volume of the extraction solution (mL); N signifies the dilution factor; M indicates the fresh weight of the citrus peel (mg).

#### 2.5.2. Total phenolic quantification.

Using gallic acid as a reference, the total phenolic content (TPC) in each extract were measured by the Folin-Ciocalteu spectrophotometric technique [33]. In brief, extract solution (1.0 mL) was mixed with 10% (v/v) Folin-Ciocatleu reagent (5.0 mL). After 5 min, 7.5% (w/v) sodium carbonate (Na_2_CO_3_) solution (4 mL) was added to the mixture. The mixture was vortexed and incubated at 25°C for 60 min. Absorbance of the solution was measured spectrophotometrically at a wavelength of 760 nm. A standard curve for gallic acid was generated with the equation Y = 7.64X + 0.0177, R² = 0.999, and the total phenolic content was calculated accordingly.

#### 2.5.3. Protein quantification.

The protein content was determined using the Coomassie Brilliant Blue method [34], with bovine serum albumin (BSA) at a concentration of 0.10 mg/mL as the standard. 1.0 mL of extract was combined with 5 mL Coomassie Brilliant Blue G-250 solution and vortexed. After 5 min, the absorbance was measured at 595 nm. A BSA calibration curve was prepared (Y = 3.8383X + 0.0376, R² = 0.997). This equation was used to calculate the protein concentration in the UPEs.

### 2.6. Targeted metabolomic analysis of flavonoids in different citrus UPEs

A 100 μL of UPE was mixed with 300 μL of the methanol solution and 10 μL of an internal standard solution (10 μg/mL). The mixture was vortexed for 30 sec, followed by sonication in a water bath at 10°C for 30 min. Subsequently, samples were centrifuged at 14,000 g for 20 min at 10°C. The resulting supernatant was filtered through a 0.22-mm membrane, and the filtrate was added to a detection bottle as a sample to be tested using UPLC-MS.

Standard solutions and sample solutions (2 µL) were analyzed on an Agilent 1290 Infinity LC ultra-high-performance liquid chromatography system and an ACQUITY UPLCR HSS T3 column (100 × 2.1 mm, 1.8 µm) maintained at 40°C. A mobile phase comprising (A) 0.1% formic acid in water(v/v) and (B)0.1% formic acid in acetonitrile (v/v) was used for gradient elution at a flow rate of 400 μL/min. The gradient program was set as follows: 0–3 min, 5–20% B; 3–9 min, 20–45% B; 9–11 min, 45–95% B; 11–13 min, 95% B; 13–13.1 min, 95–10% B; and 13.1–15 min, 10% B.

Mass spectrometric detection of metabolites was performed on a 5500 QTRAP mass spectrometer (SCIEX) with an Electrospray ion source (ESI) ion source. The parameters were as follows: ESI was used, the scanning mode was positive and negative ion switching to obtain sensitive and stable responses, the multiple reaction monitoring mode was used for detection to enhance the selectivity of detection, the source temperature was 550°C, the ion source Gas1 was 55, the ion source Gas2 was 50, the curtain gas was 30, the ion spray voltage floating was 5500 V and −4500 V for ESI (+) and ESI (−), respectively.

### 2.7. Antioxidant activity assays

#### 2.7.1. Hydroxyl radical scavenging activity.

The ·OH radical scavenging capability was measured using a previously described method with minor modifications [35]. An FeSO_4_ solution (2.0 mL, 6 mM), a salicylic acid-ethanol solution (5.0 mL, 6 mM), the sample (5.0 mL), and H_2_O_2_ (2.0 mL, 0.06% v/v) were mixed and reacted as the Fenton system. After shaking, the mixture was incubated at 37°C for 15 min. The absorbance was measured at 510 nm, and the ·OH scavenging activity was calculated using the following equation:


·OHscavengingactivity(%)=[1–(A1–A2)÷A0]×100 (3)

where A_1_ was the absorbance of the sample solution, A_2_ was the absorbance when distilled water was used in place of the salicylic acid-ethanol solution, and A_0_ was the absorbance when distilled water was used in place of the sample solution.

#### 2.7.2. Superoxide anion scavenging activity.

The O_2_^·–^ scavenging activity of the upper phase solution was assayed using the method described by Chen et al. [36,37], with slight modifications. For this, 4.5 mL of Tris-HCl buffer (50 mM, pH 8.2), 5.0 mL of sample, and 0.4 mL of catechol solution (25 mM, pre-warmed at 37°C before use) were mixed. The mixtures were vortexed and incubated at 25°C for 4 min. Then, 7.5 μL HCl (8 mM) was added to the mixture to stop the reaction. The absorbance of the UPEs was measured at 325 nm, and the O_2_^·–^ scavenging activity was calculated as follows:


$$\text{O}\text{2}\cdot^{–}\text{scavenging}\text{activity}(\%)=[\text{1}–(\text{A}\text{1}–\text{A}\text{2})÷\text{A}\text{0}]\times \text{100}$$ (4)

where A_1_ was the absorbance of the sample solution, A_0_ was the absorbance when deionized water was used in place of the sample solution, and A_2_ was the absorbance when deionized water was used in place of the catechol solution.

#### 2.7.3 ABTS radical scavenging activity.

The method described in Chen et al. [16]. was used with simple modifications. ABTS+ reagent was prepared as follows: Mix 7.4 mmol/L ABTS solution and 2.6 mmol/L potassium persulfate solution at a ratio of 1:1 (v/v), and the mixture was placed in the dark for 16 h. Each mixture was then diluted with phosphate buffer until the absorbance value reached 0.70 ± 0.02 before use. 1.0 mL of each sample was added to 1.5 mL of ABTS+ free radical solution, mixed well, and placed in the dark for 8 min. The absorbance was measured at 734 nm, and the ABTS scavenging activity was calculated using the following equation:


ABTSscavengingactivity(%)=[1–(A1–A2)÷A0]×100 (5)

where the absorbance value of a sample when ABTS+ was added is A_1_, the absorbance value when the ABTS+ solution was replaced by an equal volume of phosphate buffer is A_2_, and the absorbance value when the sample was replaced by an equal volume of phosphate buffer is A_0_.

### 2.8. Cell viability assay

The cells were cultured at 37°C in a 5% CO_2_ atmosphere in Dulbecco’s modified Eagle’s medium (DMEM) containing 10% (v/v) fetal bovine serum (FBS) and 1% antibiotics (penicillin/streptomycin, v/v). To determine the viability of HaCaT and BJ cells, cells were seeded in a 96-well plate (1×10^5^ cells/well), and incubated for 24 h at 37°C in the presence of 5% CO_2_. After 24 h of pre-culture, the medium was aspirated, and varying concentrations (0.06, 0.25, and 1.00%) of the UPEs were added to each well, and the cells were then cultured for another 24 h. Untreated cells maintained in culture medium were used as the control. Following incubation, the culture medium was decanted, and 100 μL of serum-free DMEM plus 10 μL of the CCK-8 test solution was added to all wells. The cells were incubated for 1 h, and the absorbance at 450 nm was determined using a microplate reader. Cell viability was calculated using the following formula:


Cellviability(%)=A1÷A2×100 (6)

where A_1_ was the absorbance of the sample well, and A_2_ was the absorbance of the control well.

### 2.9. Statistical analysis

All analyses were performed in triplicate (n = 3) and are reported as mean ± SD Statistical evaluation was carried out with SPSS software and GraphPad Prism 8.0 for Windows. Significant differences were tested by ANOVA and Duncan’s multiple range tests. The antioxidant activity of citrus fruits was evaluated by APC index [38]. The APC index was calculated by assigning all assays an equal weight, assigning an index value of 100 to the best score for each test, and then calculating an index score for all other samples within the test as follows: antioxidant index score= [(sample score/best score) × 100]; the average of all three tests for each UPE was then taken for the antioxidant potency composite index. PCA, OPLS-DA and HCA was performed using Wekemo Bioinloud (https://www.bioincloud.tech/).

## 3. Results and discussion

### 3.1. The Main Active Ingredient Contents in the UPEs

UPEs rich in bioactive flavonoids were obtained from citrus peels with UA-ATPE. As illustrated in Fig. S1 (Fig S1 in S1 file), the color of the solutions derived from different citrus peels darkened markedly after UA-ATPE treatment. This suggests that a substantial amount of flavonoid compounds was successfully extracted from the peels into the solution. Hereafter, the UPEs derived from Guangxi mandarin orange, Meizhou Shatian pomelo, Sichuan lemon, and Jiangxi Gannan navel orange are designated GX, MZ, SC, and JX, respectively.

**Fig 1:**
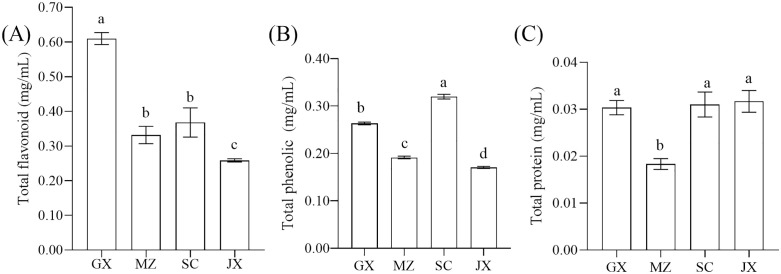
Comparison of the contents of different citrus UPEs. Note: (A) total flavonoid content; (B) total phenolic content; (C)total protein. GX: upper phase extract of Guangxi citrus peel; MZ: upper phase extract of Meizhou shatian pomelo peel; SC: upper phase extract of Sichuan lemon peel; JX: upper phase extract of Jiangxi Gannan navel orange peel. The different letters on the same bar show a significant difference according to Duncan’s test at *p* < 0.05.

Our study revealed that the total flavonoid content in the UPEs derived from citrus peels was significantly higher than the total phenolic and protein concentrations (Fig 1). Among the four UPEs, GX exhibited the highest total flavonoid content (0.61 ± 0.01 mg/mL), which was 184.00%, 139.70%, and 236.00% higher than that of MZ, SC, and JX, respectively. This finding aligns with the results of Chen et al. [23], indicating that the flavonoid content in citrus peels is higher than that in oranges, grapefruits, and lemons. Regarding total phenolic content, SC exhibited the highest concentration (0.32 ± 0.00 mg/mL), which was 1.23-fold, 1.68-fold, and 1.88-fold higher than that of GX, MZ, and JX, respectively. These results are consistent with prior research indicating that lemon peels are particularly rich in phenolic compounds [39,40].

It is well known that flavonoids are the predominant class of phenolic substances found in almost all plants. However, the total flavonoid content in this study was found to be higher than the TPC, aligning with the findings of Gómez-Mejía et al. [26]. This discrepancy might be due to several factors. First, rutin was used as the calibration standard for flavonoid quantification, which exhibits lower absorbance signals compared to quercetin across all wavelengths, resulting in higher TFC values [41]. Second, different phenolic compounds have varied reactivities in the Folin-Ciocalteu method, potentially leading to an underestimation of TPC. This variability stems from differences in the structural characteristics of these compounds, affecting their ability to reduce the Folin-Ciocalteu reagent effectively [42]. Furthermore, the presence of proteins can also affect the accuracy of phenolic compound quantification results. As suggested by related studies [43], polyphenols can form complexes with proteins, which may alter color responses or introduce quantitative errors. In this study, except for MZ (0.02 ± 0.00 mg/mL), the protein content in the other three UPEs showed no significant difference (*p* > 0.05), with a concentration of approximately 0.03 mg/mL. This limitation underscores the importance of our subsequent targeted metabolomics analysis (Section 3.3) for obtaining precise quantification of individual flavonoid metabolites.

The spectrophotometric methods (Folin-Ciocalteu for phenolics, AlCl₃ complexation for flavonoids, and Coomassie Brilliant Blue method for proteins) provided preliminary quantification of bioactive components across citrus varieties, with calibration curves demonstrating robust linearity (*R*^2 ^≥ 0.997). While these methods enabled reliable relative comparisons of inter-cultivar differences, three systematic limitations were identified: (1) Endogenous compounds caused matrix interference; (2) Standard-dependent variability emerged from differential reactivity between calibration references; (3) Unquantified accuracy errors persisted due to absent spike recovery tests. Therefore, to achieve precise measurements of these compounds, further purification and removal of contaminants, measure more quantitative calibration standards or the use of more precise analytical techniques, is necessary. Since this study primarily focuses on flavonoids, subsequent analyses will be limited to the quantification of flavonoid compounds using liquid chromatography-mass spectrometry.

### 3.2. Extraction yield of flavonoids and pectin

**Fig 2:**
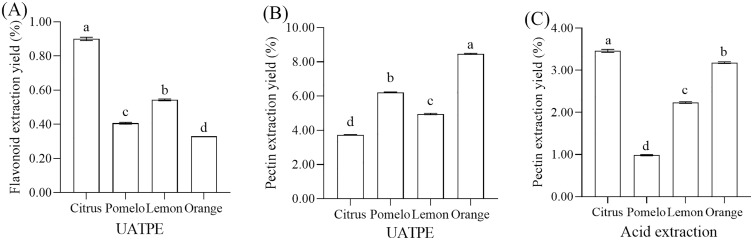
The extraction yields of flavonoids and pectin from different citrus peels. (A) Flavonoid extraction yield; (B) Pectin extraction yield. UA-ATPE: Ultrasound-assisted aqueous two-phase extraction; Citrus: Guangxi mandarin orange peel; Pomelo: Meizhou shatian pomelo peel; Lemon: Sichuan lemon peel; Orange: Jiangxi Gannan navel orange peel. The different letters on the same bar show a significant difference according to Duncan’s test at *p* < 0.05.

Under the same extraction conditions, different citrus peels not only exhibited variations in the content of active compounds but also showed differences in the volume changes after extraction (Table S1 in S1 File). This ultimately led to significant differences in the extraction yields of the active compounds (Table S2 in S1 File). As shown in Fig 2A and 2B, after UA-ATPE, citrus peels of different varieties yield varying contents of flavonoids in the upper phase and pectin in the lower phase (*p* < 0.05). Among these (Fig 2A), Guangxi mandarin orange exhibited the highest flavonoid extraction yield at 0.90 ± 0.00%, which was 2.25, 1.67, and 2.73 fold higher than Meizhou shatian pomelo, Sichuan lemon, and Jiangxi Gannan navel orange, respectively. The optimized ultrasonic-assisted extraction protocol achieved total flavonoid yields of 0.54 ± 0.00% (lemon peel) and 0.33 ± 0.00% (orange peel), representing 41.7% and 13.8% increases respectively over values reported by Özcan et al. [44] for analogous citrus varieties using conventional extraction techniques. These discrepancies are likely due to differences in citrus varieties, extraction conditions, and solvents. The total flavonoid content of pomelo peel extract obtained by enzyme- and ultrasound-assisted extraction is 10.69 mg RE/g [45]. Long et al. [46] employed a combination of supercritical fluid extraction (SFE) and continuous high-speed counter-current chromatography (HSCCC) to extract flavones from Citri Reticulatae Pericarpium (CRP), achieving a extraction yield of 2.08 mg/g. Their result was significantly lower than that obtained by the method utilized in this study. Our method yields higher flavonoid contents: GX (30.03 mg/g dw), MZ (17.98 mg/g dw), SC (36.90 mg/g dw), and JX (12.32 mg/g dw). UA-ATPE achieves 17.98 mg/g dw flavonoids from pomelo peel, eliminating enzyme dependency and enabling pectin co-recovery for superior industrial scalability.

The extraction efficiency of flavonoids from citrus peels is significantly influenced by factors such as peel thickness, structural characteristics, moisture content, and intercellular spaces [47]. For instance, thick-skinned varieties like pomelo typically contain higher levels of pectin and cellulose. This leads to increased cell wall density and, consequently, reduced extraction efficiency. In contrast, thin-skinned varieties like mandarins and oranges possess relatively loose cellular structures, which facilitate the release of bioactive compounds and resulting in higher extraction rates. Furthermore, increased moisture content enhances solvent penetration, further improving extraction efficiency [22]. Interestingly, the flavonoid extraction rate from lemons is higher than that from thinner-skinned citrus varieties such as mandarins and oranges. This is likely due to the elevated moisture content in Sichuan lemons. Thus, the flavonoid extraction yield from lemons is higher than that from thinner-skinned citrus varieties such as mandarins and oranges. This difference may be attributed to the higher moisture content in lemons (85.24 ± 0.03%) compared to mandarins (69.58 ± 0.00%) and oranges (73.13 ± 0.00%) (Table 1).

To evaluate whether the UA-ATPE method is more suitable for industrial production compared to acid-assisted extraction, we compared the extraction yields of the two methods. In the UA-ATPE method (Fig 2B), the pectin extraction yield of Jiangxi Gannan navel orange is significantly higher than that of Meizhou shatian pomelo, Sichuan lemon, and Guangxi mandarin orange, with extraction yield of 8.47%, 6.22%, 4.96%, and 3.73%, respectively. As shown in Fig 2C, except for Guangxi mandarin orange, where the two methods showed no significant difference in extraction yield, in the other three citrus peels, the pectin extraction yield with UA-ATPE was significantly higher than that with hydrochloric acid extraction. This indicates that ultrasound-assisted aqueous two-phase extraction of pectin is more efficient and more suitable for industrial-scale extraction. For the extraction yield, the pectin extraction yield ranged from 0.98%–3.46% with hydrochloric acid extraction, while it ranged from 3.73%−8.47% with UA-ATPE. In our study, the extraction efficiency of pectin using acid-assisted extraction and UA-ATPE was significantly lower compared to the findings of Martyna et al. [33], whose acid-assisted extraction yield ranged from 7.6% to 17.6%, while the yield for ultrasonic-assisted extraction ranged from 9.9% to 28.2%. Our results were far below this range, and these differences probably ascribed the different extraction method details, region of production, cultivar and stage of ripeness.

Although UA-ATPE enables the green and efficient co-extraction of flavonoids and pectin from citrus peel, this study was conducted under controlled laboratory conditions. This highlights a key limitation regarding the translation of our findings to industrial-scale operations. Practical challenges such as solvent recovery, process continuity, energy consumption, and cost-efficiency remain to be addressed before large-scale implementation becomes viable. Moreover, for flavonoid-rich extracts to be successfully applied in food, cosmetics, or pharmaceuticals, their physicochemical stability must be ensured under processing and storage conditions. Previous studies have shown that flavonoid stability is significantly influenced by environmental factors. For instance, glycosylated flavonoids such as naringin and hesperidin exhibit relatively high thermal stability, while aglycone forms are more prone to degradation at elevated temperatures [48]. Similarly, flavonoids are generally stable at acidic to neutral pH but tend to degrade in alkaline environments [49].

Therefore, future work must therefore address both the technical and economic barriers to industrial UA-ATPE scale-up and the stability of the resulting extracts under diverse physical–chemical conditions to guarantee consistent functionality in end-use formulations.

### 3.3. Targeted metabolomic analysis of flavonoids in different citrus varieties

#### 3.3.1. Multivariant chemometric analysis.

**Fig 3:**
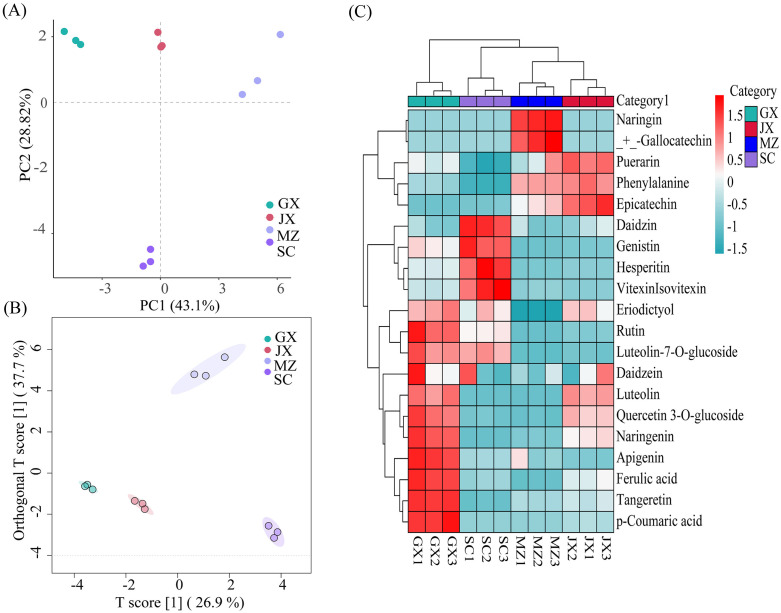
The plot of (A) PCA, (B) OPLS-DA scores and (C) Heatmap for different UPEs. Note: heatmap showing the distribution and concentration of the top twenty flavonoid compounds. Red boxes mean concentrations are higher among different fruit peel samples. Blue boxes mean lower concentrations. Different clusters of samples indicate significant differences in flavonoids profiles. GX: upper phase extract of Guangxi mandarin orange peel; MZ: upper phase extract of Meizhou shatian pomelo peel; SC: upper phase extract of Sichuan lemon peel; JX: upper phase extract of Jiangxi Gannan navel orange peel.

To elucidate the metabolic heterogeneity across geographically distinct citrus varieties, we systematically quantified 40 characteristic metabolites in peel extracts from the four selected cultivars using a targeted metabolomics approach. Principal component analysis (PCA) and hierarchical cluster analysis (HCA) are commonly used for classifying samples into different groups with different algorithms [50,51]. PCA was first used for preliminary analysis to observe trends in group aggregation and separation, as well as to visualize the similarities or differences among the UPEs from different citrus varieties. The two principal components (PC1and PC2) together explain 71.92% of the total variance (43.10% and 28.82%, respectively). As shown in Fig 3A, all samples were classified into four groups corresponding to their metabolites, which further demonstrates that the citrus varieties have a significant effect on the metabolome. The chemical contents of UPEs from different citrus varied.

Additionally, we employed a more precise supervised orthogonal partial least squares discriminant analysis (OPLS-DA) to enhance the accuracy of our analysis. OPLS-DA offers better classification efficiency than the PCA model, which enables the filtering of system noise and extraction of variable information [52]. As shown in Fig 3B, better separation was obtained between groups, samples marked in the same colour were tightly close to each other, keeping away from other classes, which indicates that the UPEs from different citrus varied could be completely distinguished. R^2^Y and Q^2^ are used to assess the validity of the model [53]. Multiple permutation tests (n = 1000) were performed to generate different random Q^2^ values, further testing the model’s robustness. The values of R^2^Y = 0.987 and Q^2 ^= 0.975 (Fig S2 in S1 File) were all close to 1, indicating the excellent capability and predictability of the model.

For further analyzing the hierarchical clustering of targeted flavonoid compounds in the UPE from different citrus varieties, a HCA map was constructed. As shown in Fig 3C, different clusters of samples indicate significant differences in flavonoids profiles. The color difference showed the abundance of flavonoids in different fruit peels. From the HCA map, it can be observed that JX, MZ and GX were clustered together in the group, which shared similar patterns of flavonoid contents. Flavonoid compounds were also grouped into five main clusters in the dendrogram. Overall, these clusters indicated that flavonoids had greater similarity in terms of the concentration among different fruit peel samples. However, some flavonoids (naringin and (+)-gallocatechin) showed variability with respect to other flavonoids compound clusters.

Multivariate analyses (PCA, OPLS-DA, and HCA) robustly distinguished metabolomic profiles of citrus peel extracts (UPEs) across four varieties, with PCA explaining 71.92% variance and OPLS-DA achieving high model validity (R^2^Y = 0.987, Q^2^ = 0.975). Hierarchical clustering revealed species-specific flavonoid patterns, grouping three regional varieties (JX, MZ, GX) with shared compositional traits, while identifying distinct flavonoid subclusters (e.g., naringin, (+)-gallocatechin) that drive inter-variety differentiation. These findings validate the combined influence of geographical origin and citrus variety on metabolite diversity, offering a statistical basis for region-specific valorization of citrus by-products.

#### 3.3.2. Identification and screening of differential metabolites.

**Fig 4:**
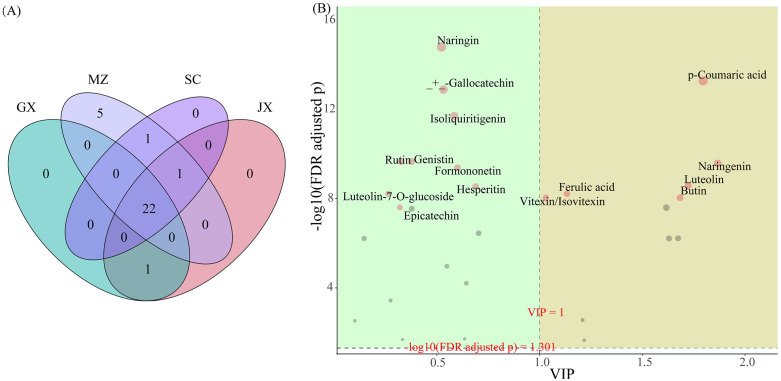
Differential metabolites in upper phase extracts from selected citrus peels. (A) Venn diagram (B) OPLS-DA. GX: upper phase extract of Guangxi mandarin orange peel; MZ: upper phase extract of Meizhou shatian pomelo peel; SC: upper phase extract of Sichuan lemon peel; JX: upper phase extract of Jiangxi Gannan navel orange peel.

A total of 37 flavonoid metabolites, 2 phenolic acids (p-coumaric acid, ferulic acid), and 1 amino acid (phenylalanine) were quantified (Table S3 and Fig S4 in S1 File). The inclusion of p-coumaric acid, ferulic acid, and phenylalanine is crucial as these compounds serve as significant intermediates or precursors in the biosynthesis of flavonoids. Comprehensive measurement of these compounds enhances our understanding of metabolic pathways. Among which ten common flavonoid metabolites, including quercetin, dihydroquercetin, myricetin, dihydromyricetin, daidzein, quercetin, isoquercetin, catechin, epicatechin, and epigallocatechin gallate, were not detected in these four UPEs. As shown in Fig 4(A), venn diagram shows the relations of metabolites present in different UPEs. Among the 40 detected metabolites, 22 were found in all four UPEs. Among them, naringin, formononetin, isoliquiritigenin, liquiritigenin, and (+)-gallocatechin were unique to the MZ. Naringin, the primary flavonoid compound in MZ, reached a high content of 27116.54 ± 1625.84 ng/mL (Table S3 in S1 File). Different species were characterized by different individual flavonoid compounds, naringin was the dominant compound in the peel of pummelo, while GX mandarin was rich in tangeretin (57434.62 ± 1052.19 ng/mL). This result is similar to that of Xi et al. [54]. Additionally, the concentration of Hesperitin in the SC and JX citrus varieties was found to be notably higher compared to the other metabolites measured. Kaempferide was detected in all UPEs except GX, but its content was less than 1.00 ng/mL. Coumarin was only detected in GX and JX, with a content in GX that was 10.08 times that in JX. On the other hand, butin was only found in MZ and SC, with both extracts having contents less than 1.10 ng/mL. Hesperidin and hesperetin, found in high concentrations in all four extracts, may cause ion suppression during LC-MS analysis, which could reduce the detection of other compounds. Furthermore, in terms of biological activity, these dominant compounds may lead to a concentration-dominant masking effects, potentially masking the contributions of other beneficial bioactive substances present in lower concentrations.

To optimize the selection of citrus varieties for efficient flavonoid extraction, we performed a statistical analysis of flavonoid metabolite yields across different varieties. As shown in Table S4 in S1 File, the yield of several metabolites in the Guangxi mandarin orange variety was notably higher than in the other three varieties. These include tangeretin (86008.35 ± 1575.65 μg/100g fw), p-coumaric acid (5098.84 ± 323.41 μg/100g fw), luteolin (4175.67 ± 434.16 μg/100g fw), rutin (4406.87 ± 600.91 μg/100g fw), ferulic acid (1405.95 ± 68.28 μg/100g fw), luteolin-7-O-glucoside (627.68 ± 70.32 μg/100g fw), Quercetin 3-O-glucoside (182.77 ± 17.14 μg/100g fw), naringenin (53.52 ± 3.51 μg/100g fw), eriodictyol (40.48 ± 3.84 μg/100g fw), apigenin (4.85 ± 0.12 μg/100g fw), genistein (3.79 ± 0.39 μg/100g fw), and puerarin (2.66 ± 0.16 μg/100g fw). In the Sichuan lemon variety, hesperitin (332949.58 ± 51129.63 μg/100g fw), vitexin/isovitexin (468.55 ± 83.7 μg/100g fw), genistin (169.17 ± 17.52 μg/100g fw), and daidzin (8.33 ± 0.54 μg/100g fw) exhibited the highest yields. Meanwhile, phenylalanine (4468.41 ± 271.43 μg/100g fw) and epicatechin (28.73 ± 2.61 μg/100g fw) were significantly more abundant in the Jiangxi navel orange variety. These findings highlight the metabolic diversity among citrus species, providing insights into the optimal selection of varieties for the targeted extraction of specific flavonoids for industrial and research purposes.

To understand the influence of the 40 measured compounds on the quality differences among various citrus species, we employed discriminant analysis using the variable importance in projection (VIP) method to determine the most important metabolites. These metabolites serve as important references for distinguishing between different UPEs. Metabolites with VIP > 1 and *p* < 0.05 are considered differential metabolites, with higher VIP values contributing more significantly to group differentiation [55]. As shown in Fig 4, p-coumaric acid, naringenin, luteolin, butin, ferulic acid, and vitexin/isovitexin were identified as the main differential metabolites in flavonoid compositions among GX, MZ, SC, and JX, with their contributions to the differentiation of these varieties decreasing in the order mentioned. This indicates that the presence and levels of these six metabolites effectively distinguish these four citrus varieties. In these four varieties of UPE, the differential metabolite butin is only present in MZ (1.09 ± 0.48 ng/mL) and SC (0.78 ± 0.09 ng/mL), with significantly higher levels in MZ compared to SC (*p* < 0.05). p-Coumaric acid is found exclusively in GX (3404.89 ± 176.34 ng/mL) and JX (337.77 ± 46.12 ng/mL), with significantly higher levels in GX than in JX (*p* < 0.05). In addition, naringenin had the highest content in GX, at 35.74 ± 2.34 ng/mL, while SC had the lowest content, at 2.63 ± 0.12 ng/mL. The content of luteolin and ferulic acid in GX was higher than that in JX, SC, and MZ. Vitexin/isovitexin had the highest content in SC, at 316.59 ± 56.55 ng/mL. The screening of differential metabolites can reveal the synergistic regulation of flavonoid biosynthesis by the genetic background of varieties and the geographical environment, and these differential metabolites are not only markers for variety identification, but also core contributors to functional activities.

### 3.4. Analysis of antioxidant capacity

**Fig 5:**
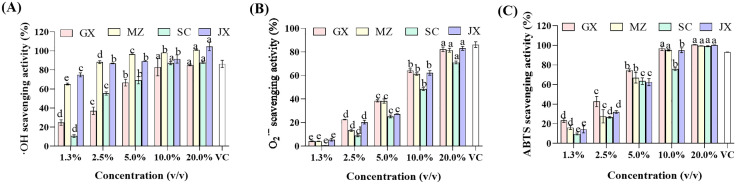
Comparison of antioxidant activities of upper phase extracts from different citrus peels. (A)·OH scavenging capacity, (B) O2·– scavenging capacity, and (C) ABTS scavenging capacity. GX: upper phase extract of Guangxi mandarin orange peel; MZ: upper phase extract of Meizhou shatian pomelo peel; SC: upper phase extract of Sichuan lemon peel; JX: upper phase extract of Jiangxi Gannan navel orange peel; VC: ascorbic acid. The different letters on the same bar show a significant difference according to Duncan’s test at *p* < 0.05.

A single method was insufficient to fully reflect the antioxidant capacity of UPEs due to their complex constituents, as different antioxidants responded differently to various tests. Therefore, the total antioxidant activities of UPEs were evaluated by three widely used in vitro chemical assays including ·OH, O_2_^·–^ and ABTS. The results as shown in Fig 5, the ·OH, O_2_^·–^ and ABTS scavenging capacity increased with UPEs concentrations. The IC_50_ values shown in Table 2 indicate significant variations in the antioxidant activities of different UPEs. It should be noted that the smaller the IC_50_ value, the stronger the scavenging ability and antioxidant capacity.

**Table 2:** Antioxidant activity of UPEs expressed as IC₅₀ values in various radical scavenging assays.

Samples	·OH (%, v/v)	O_2_^·-^ (%, v/v)	ABTS (%, v/v)	APC (%, v/v)	Rank
MZ	0.91 ± 0.01 c	7.37 ± 0.27 b	3.56 ± 0.11 b	2.64	1
JX	0.38 ± 1.15 d	7.72 ± 1.40 b	3.58 ± 0.32 b	2.92	2
GX	3.31 ± 0.39 a	6.76 ± 0.22 c	2.71 ± 0.11 c	3.69	3
SC	2.85 ± 0.07 b	10.66 ± 0.33 a	4.14 ± 0.10 a	4.81	4

Note: Averaged for all three tests for each UPE for the antioxidant potency composite index, antioxidant index score = [(sample score/best score) × 100]. GX: upper phase extract of Guangxi mandarin orange peel; MZ: upper phase extract of Meizhou shatian pomelo peel; SC: upper phase extract of Sichuan lemon peel; JX: upper phase extract of Jiangxi Gannan navel orange peel. The different letters on the same row show a significant difference according to Duncan’s test at *p* < 0.05.

The results from the ·OH scavenging capacity assessment are presented in Fig 5A and Table 2. The strongest ·OH scavenging capacity was detected is JX (IC_50_ = 0.38 ± 1.15%), followed by MZ and SC, while GX had the weakest antioxidant capacity. The ·OH radical scavenging rate of JX at a concentration of 2.5% (74.83 ± 1.69%) is not significantly different with that of ascorbic acid at 0.5 mg/mL (86.33 ± 0.03%). The content of flavonoid compounds such as puerarin, phenylalanine, and epicatechin in JX was significantly higher than that of other three UPEs, which is consistent with the results of antioxidant capacity, indicating that these compounds may play a key role in enhancing antioxidant capacity. Related studies have also demonstrated that puerarin [56], phenylalanine [57], and epicatechin [58] are natural antioxidants capable of effectively scavenging radicals. In addition, the lower ·OH radical scavenging capacity in GX could be attributed to significant differences in the distribution pattern of its flavonoid metabolites compared to the other three UPEs (Fig 3C).

The Table 2 and Fig 5B shows that the order of O_2_^·–^ radical scavenging capacity from highest to lowest was GX > MZ, JX > SC. The O_2_^·–^ radical scavenging rate of GX at its highest concentration measured is 82.00 ± 1.56%, significantly lower than that of 0.5 mg/mL ascorbic acid (86.20 ± 0.02%). Similar trends were observed in ABTS radical scavenging (Fig 5C). Among the four UPEs, GX exhibited the highest scavenging rate, while SC showed the lowest scavenging rate. The ABTS radical scavenging rate of GX at a concentration of 10.0% (96.86 ± 1.77%) is not significantly different from that at 0.5 mg/mL ascorbic acid (93.19 ± 0.00%). Different citrus UPE showed different antioxidant activity, which might be due to the structure and type of antioxidants detected in the extracts of citrus peels.

It is interesting that GX, which had significant O_2_^·–^ and ABTS radical inhibitory activity, showed the lowest scavenging ·OH capacity. This indicates that evaluating antioxidant capacity cannot rely solely on a single indicator; multiple factors need to be considered comprehensively. To enable a comprehensive comparison of the overall antioxidant activities among different UPEs, we calculated the antioxidant potency composite (APC) index for each variety using the method described by Seeram et al. [38]. As the ranking of APC indices varies at different sample concentrations (Fig S3 in S1 File), we computed the sample concentration at which the APC index is 50%. A lower sample concentration indicates stronger antioxidant capacity. The order of antioxidant potency for UPEs was as follows: MZ > JX > GX > SC.

### 3.5. Correlation analysis between major active ingredients and antioxidant activity

To determine whether the differential metabolites identified above account for the superior antioxidant activity of the MZ extract, Pearson correlation coefficients were calculated between its antioxidant indices and the concentrations of individual active constituents (Table 3), on the premise that the antioxidant capacity of fruit extracts is governed by these compounds ABTS radical scavenging was based on the SET mechanism [59], while ·OH radical scavenging may involve both proton transfer and single electron transfer mechanisms to eliminate free radicals [60]. Due to the negative correlation (r = – 0.999) between the IC_50_ of ABTS radical scavenging activity and ·OH radical scavenging activity, it can be inferred that the ·OH radical scavenging activity of MZ is achieved through proton transfer.

**Table 3:** Correlation analysis between components and major differential metabolites of MZ and antioxidation.

	TF	TP	Protein	Nar	Luteolin	Butin	V/I	FA	·OH IC_50_	O_2_^·–^IC_50_	ABTS IC_50_	APC IC_50_
TF	1											
TP	0.971	1										
Protein	−0.971	−1.000^**^	1									
Nar	0.835	0.679	−0.679	1								
Luteolin	−0.839	−0.684	0.684	−1.000^**^	1							
Butin	0.966	1.000^*^	−1.000^*^	0.664	−0.669	1						
V/I	−0.950	−0.997^*^	0.997^*^	−0.623	0.629	−.999^*^	1					
FA	−0.927	−0.990	0.990	−0.567	0.573	−0.992	0.998^*^	1				
·OH IC_50_	−0.704	−0.854	0.854	−0.197	0.204	−0.864	0.890	0.919	1			
O_2_^·–^ IC_50_	0.216	−0.025	0.025	0.717	−0.712	−0.045	0.098	0.167	0.542	1		
ABTS IC_50_	0.738	0.878	−0.878	0.245	−0.252	0.888	−0.911	−0.937	−.999^*^	−0.500	1	
APC IC_50_	0.994	0.939	−0.939	0.889	−0.892	0.932	−0.912	−0.881	−0.624	0.319	0.661	1

TF: total flavonoid; TP: total phenolic; FA: ferulic acid; Nar: Naringenin; V/I: vitexin/isovitexin; ** 0.001 < *p* < 0.01.

Numerous studies have shown a correlation between the content of phenolic compounds and flavonoids in citrus peels and their antioxidant activity [61,62]. However, this study found no significant correlation between the total phenolic and total flavonoid content in MZ and its free radical scavenging activity. This result is consistent with the findings of Ghasemi et al. [63], who also observed no significant correlation between the total phenolic and/or flavonoid content and antioxidant activity in tissues and/or peels. This discrepancy may be due to the selective nature of the methods used for determining these compounds [64]. For example, the aluminum chloride’s selective reaction with flavonols and the flavone luteolin [65], leading to an inaccurate measurement of flavonoid content, which in turn affects the observed correlation between total flavonoids and antioxidant activity. In addition, the identified differential metabolites also showed no significant correlation with the three types of free radicals. This indicates that these differential metabolites are not the primary factors leading to MZ having stronger antioxidant activity than the other three UPEs. Relevant studies suggest that naringin [66,67], formononetin [64], isoliquiritigenin [68], liquiritigenin [69], and (+)-gallocatechin [69] exhibit significant scavenging activities against hydroxyl and superoxide radicals. These five components are unique to MZ compared to the other three UPEs. Therefore, it is hypothesized that MZ’s superior overall antioxidant capacity (APC) compared to other UPEs may be significantly attributed to the presence and efficient extraction of these five unique components (naringin, formononetin, isoliquiritigenin, liquiritigenin, (+)-gallocatechin). The UA-ATPE process likely facilitates the effective recovery of these specific bioactive compounds, which might be less efficiently extracted or degraded under the conditions employed in harsher traditional methods

As shown in Table 3, vitexin/isovitexin content exhibits multiple correlations with other components content. Specifically, vitexin/isovitexin content is significantly negatively correlated with total phenolic (r = – 0.997) and butin content (r = – 0.999), and positively correlated with protein content (r = 0.997) and ferulic acid content (r = 0.998). Total phenolic content shows significant negative correlations not only with vitexin/isovitexin content (r = – 0.997) but also with protein content (r = – 1.000), while exhibiting a significant positive correlation with butin content (r = 1.000). Additionally, naringenin content and luteolin content also show a significant negative correlation (r = – 1.000). These components are crucial in the flavonoid metabolic process, and their interrelationships may influence flavonoid metabolism. Among them, the content of vitexin/isovitexin shows significant correlations with the other four components, suggesting it might have the most significant impact on the flavonoid metabolic pathways. Besides the antioxidant capacity of natural phytochemicals themselves, interactions or synergies among various active ingredients may also contribute to their antioxidant abilities. For example, the flavonols, quercetin and quercetin-3-glucoside trigger a noticeable increase in antioxidant activity when mixed in solution with another flavonoid [70]. Furthermore, proteins and polyphenols can interact through non-covalent bonds (hydrophobic, ionic, and hydrogen bonds) or covalent bonds, thereby enhancing the antioxidant properties of conjugates [71].

The superior antioxidant capacity of MZ may primarily arise from the synergistic or cooperative interactions among its unique components. To determine whether the five specific compounds unique to MZ are responsible for its superior antioxidant activity compared to the other three varieties, it is essential to isolate and purify these five compounds. Subsequently, we will evaluate their individual and combined antioxidant capacities, as well as their combined antioxidant activity, to ascertain their potential contributions to the overall antioxidant efficacy of MZ. Additionally, other antioxidant compounds, such as ascorbic acid, might also play a significant role. Despite the notable degradation of ascorbic acid during the 80°C ultrasonic extraction process [72], the residual amount of ascorbic acid and its contribution to antioxidant activity remain unclear. Therefore, further measurement of ascorbic acid content is necessary to fully understand its impact.

### 3.6. Effect of UPEs on skin cell viability

In this study, cell viability was quantified using the CCK-8 assay, which relies on the reduction of WST-8 by cellular dehydrogenases in viable cells, producing a water-soluble formazan dye. The intensity of the resulting color, measured via absorbance at 450 nm, is directly proportional to the number of metabolically active cells, offering a reliable indicator of cell health.

**Fig 6:**
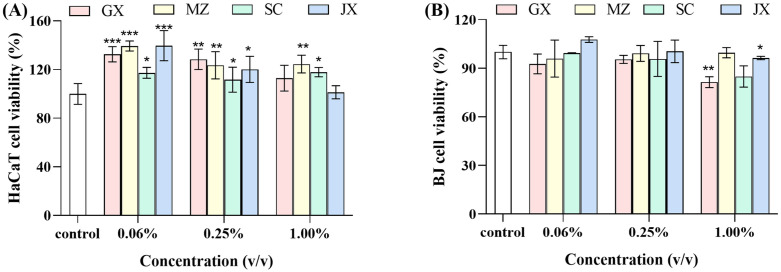
Effects of upper phase extracts from different citrus peels on cell viability. Note:(A) HaCaT cell viability; (B) BJ cell viability. GX: upper phase extract of Guangxi mandarin orange peel; MZ: upper phase extract of Meizhou shatian pomelo peel; SC: upper phase extract of Sichuan lemon peel; JX: upper phase extract of Jiangxi Gannan navel orange peel. Each value represents the mean ± standard error mean (n = 3). * *p* < 0.01, ** *p* < 0.001, *** *p* < 0.0001 versus control (analyzed by means of two-way ANOVA).

To assess the effects of four citrus peel extracts on human skin cells, human keratinocytes (HaCaT) and fibroblasts (BJ) were tested for cell viability using the CCK-8 assay method. Fig 6A shows that HaCaT cells treated with different citrus peel UPEs exhibited over 100% cell viability, indicating that the UPEs positively affected the viability of HaCaT cells in vitro. In general, cell viability decreased with increasing concentrations of citrus peel extracts [73]. However, the cellular viability of SC did not exhibit significant variations across different concentrations (*p* > 0.05), suggesting that within the tested concentration range, cellular responsiveness to SC remains relatively consistent. Among them, when the concentration of UPE from different citrus peels ranged from 0.06% to 0.25%, all UPEs significantly promoted cell proliferation activity. At a concentration of 1.00%, MZ significantly enhanced the viability (124.49 ± 5.91%) of HaCaT cells compared to the other three UPEs. Similar effects were reported by Chen et al. [74] on HepG2 cells after contact with fresh Citrus sinensis peel extracts. The cytoprotective effect observed is most probably attributable to the levels of bioactive flavonoids, mainly naringin, puerarin, phenylalanine, and tangeretin, among others. Due to lower levels of these constituents in the SC compared to the other three UPEs, its cellular activity was significantly lower at a sample concentration of 0.06%. Studies have shown that these flavonoid compounds exhibit protective effects on cells. For example, Ly et al. [75] demonstrated that puerarin promotes keratinocyte proliferation and migration, thereby facilitating the recovery of dexamethasone-induced wound healing impairment in both in vitro and in vivo models. Zhu et al. [76]showed that naringenin protected HaCaT cells from UVB-induced damage by inhibiting TRPV1 and its phosphorylated form, which reduced apoptosis and promoted cell proliferation.

The UPEs significantly reduced the cell viability of BJ cells compared to that of HaCaT cells. According to the International Standard ISO 10993−5 (2009), reducing cell viability by more than 30% is considered a cytotoxic effect [77]. The results (Fig 6B) show that BJ cell treated with different citrus peel UPEs had over 80% cell viability. Therefore, none of the UPEs were shown to exert a cytotoxic effect at any of the concentrations used. In addition, at concentrations of 0.25–1.00%, the UPEs from different citrus varieties showed no significant difference in BJ cell viability (*p* > 0.05). However, at 1.00%, the UPE from GX (81.43 ± 2.73%) exhibited lower cell viability in BJ cells compared to the cell viability observed with JX (96.29 ± 0.85%). The 1.00% concentration of GX may induce cellular imbalance in both intra- and extracellular environments, subsequently impacting normal cellular metabolism and physiological functions, thereby resulting in decreased cellular viability. The above results show that the influence of the UPEs on the viability of HaCaT (Fig 6A) and BJ (Fig 6B) depends on both the type of extract and the concentration used [78]. While cell-based validation of UPE safety provides valuable insights, it does not comprehensively confirm its safety. The next step requires in vivo validation using animal models, such as mice, to fully assess safety.

## 4. Conclusions

In this study, ultrasonic-assisted aqueous two-phase extraction (UA-ATPE) was used to extract flavonoid-rich compounds from the peels of four citrus varieties sourced from different regions. The flavonoid content and antioxidant activity of the UPEs were analyzed, revealing significant varietal differences. Multivariate statistical analyses, including principal component analysis (PCA), orthogonal partial least squares discriminant analysis (OPLS-DA), and hierarchical cluster analysis (HCA), identified six key differential metabolites: p-coumaric acid, naringenin, luteolin, butin, ferulic acid, and vitexin/isovitexin.

The study found that the GX had higher flavonoid extraction yield and content, as well as superior superoxide anion and ABTS radical scavenging capacities compared to the other three upper phase extracts (UPEs). However, its hydroxyl radical scavenging capacity was significantly lower than the other three UPEs. This suggests that the total flavonoid content in GX does not significantly affect ·OH radical scavenging capacity. Evaluating the overall antioxidant capacity of the four UPEs based on IC_50_ values, the antioxidant capacity ranking was MZ > JX > GX > SC. Analysis of MZ’s antioxidant superiority in relation to differential metabolites revealed no significant correlation between differential metabolites and antioxidant indices. Due to relevant studies indicating significant antioxidant capabilities of naringin, formononetin, isoliquiritigenin, liquiritigenin, and (+)-gallocatechin, it is hypothesized that the antioxidant advantage of MZ may be associated with these unique components specific to MZ compared to the other three UPEs. Furthermore, cell viability assessments on HaCaT and BJ cells indicated that none of the four UPEs adversely affected cell viability. Future research will focus on expand the scope to include a wider range of citrus species, identifying key antioxidant active ingredients, elucidating specific mechanisms, and confirming their safety and non-toxicity in animal models. In conclusion, this study presents the flavonoid metabolic profiles of four citrus varieties and speculates on five related metabolites with potential antioxidant properties, providing new insights for the globe industrial utilization of flavonoid resources from citrus peels.

## Supporting information

S1 File Fig S1. Changes in the physical appearance of citrus peels from various cultivars following ultrasound-assisted aqueous two-phase extraction (UA-ATPE). Note: (A), (B), (C), and (D) represent the solutions of Guangxi mandarin orange peel, Meizhou shatian pomelo peel, Sichuan lemon peel, and Jiangxi navel orange peel without UA-ATPE treatment, respectively; (E), (F), (G), and (H) represent the solutions of Guangxi citrus peel, Meizhou shatian pomelo peel, Sichuan lemon peel, and Jiangxi navel orange peel with UA-ATPE treatment, respectively. **Fig S2.** A plot of the random distribution of test statistic Q2 values for the OPLS-DA permutation test. **Fig S3.** Comparison of antioxidant potential composite (APC) indices of different citrus peel upper phase extracts. GX: upper phase extract of Guangxi mandarin orange peel; MZ: upper phase extract of Meizhou shatian pomelo peel; SC: upper phase extract of Sichuan lemon peel; JX: upper phase extract of Jiangxi Gannan navel orange peel. **Fig S4.** Total ion chromatograms of upper phase extracts from four different citrus peels. GX: upper phase extract of Guangxi citru peel; MZ: upper phase extract of Meizhou shatian pomelo peel; SC: upper phase extract of Sichuan lemon peel; JX: upper phase extract of Jiangxi Gannan navel orange peel. **Table S1.** Volumes of upper and lower phases with UA-ATPE treatment of different citrus peels. **Table S2.** Total flavonoid, total phenol, and protein extraction yields in four varieties of citrus peels. **Table S3.** Contents of forty flavonoids determined in different UPEs. **Table S4.** Contents of forty flavonoids determined in four citrus-peel varieties. **Table S5.** Comparison of the total flavonoid, total phenolic and protein contents of UPEs from four citrus varieties. **Table S6.** The extraction yields of pectin from four different citrus peels. **Table S7.** Comparison of antioxidant activities of UPEs from selected citrus peels. **Table S8.** Effects of UPEs from selected citrus peels on HaCaT cell viability. **Table S9.** Effects of UPEs from different citrus peels on BJ cell viability.
(DOC)

## Abbreviations

UPE: upper phases extract
GX: the UPE of Guangxi mandarin orange
MZ: the UPE of Meizhou shatian pomelo
SC: the UPE of Sichuan lemon
JX: the UPE of Jiangxi Gannan navel orange
OH: hydroxyl radical
O2·−: superoxide anion
APC: antioxidant potential composite
UA-ATPE: Ultrasound-assisted aqueous two-phase extraction
EIS: Electrospray ion source
BSA: bovine serum albumin
DMEM: Dulbecco’s modified Eagles medium
FBS: fetal bovine serum
CCK8: Cell Counting Kit-8
PCA: principal component analysis
HCA: hierarchical cluster analysis
OPLS-DA: orthogonal partial least squares discriminant analysis
VIP: variable importance in projection.
