# Supplementary material for: Metabolomic profiling, antioxidant activity, and skin cell viability of citrus peel flavonoids extracted via ultrasonic-assisted aqueous two-phase system
Source: PLoS One. 2025 Dec 12;20(12):e0336325. doi: 10.1371/journal.pone.0336325 (PMC12700371; doi:10.1371/journal.pone.0336325)
Supplement: S1 File — Changes in the physical appearance of citrus peels from various cultivars following ultrasound-assisted aqueous two-phase extraction (UA-ATPE). Note: (A), (B), (C), and (D) represent the solutions of Guangxi mandarin orange peel, Meizhou shatian pomelo peel, Sichuan lemon peel, and Jiangxi navel orange peel without UA-ATPE treatment, respectively; (E), (F), (G), and (H) represent the solutions of Guangxi citrus peel, Meizhou shatian pomelo peel, Sichuan lemon peel, and Jiangxi navel orange peel with UA-ATPE treatment, respectively. Fig S2. A plot of the random distribution of test statistic Q2 values for the OPLS-DA permutation test. Fig S3. Comparison of antioxidant potential composite (APC) indices of different citrus peel upper phase extracts. GX: upper phase extract of Guangxi mandarin orange peel; MZ: upper phase extract of Meizhou shatian pomelo peel; SC: upper phase extract of Sichuan lemon peel; JX: upper phase extract of Jiangxi Gannan navel orange peel. Fig S4. Total ion chromatograms of upper phase extracts from four different citrus peels. GX: upper phase extract of Guangxi citru peel; MZ: upper phase extract of Meizhou shatian pomelo peel; SC: upper phase extract of Sichuan lemon peel; JX: upper phase extract of Jiangxi Gannan navel orange peel. Table S1. Volumes of upper and lower phases with UA-ATPE treatment of different citrus peels. Table S2. Total flavonoid, total phenol, and protein extraction yields in four varieties of citrus peels. Table S3. Contents of forty flavonoids determined in different UPEs. Table S4. Contents of forty flavonoids determined in four citrus-peel varieties. Table S5. Comparison of the total flavonoid, total phenolic and protein contents of UPEs from four citrus varieties. Table S6. The extraction yields of pectin from four different citrus peels. Table S7. Comparison of antioxidant activities of UPEs from selected citrus peels. Table S8. Effects of UPEs from selected citrus peels on HaCaT cell viability. Table S9. Effects [file pone.0336325.s001.doc]

**Figs （S1-S4)**





Fig S1. Changes in the physical appearance of citrus peels from various cultivars following ultrasound-assisted aqueous two-phase extraction (UA-ATPE). (A), (B), (C), and (D) represent the solutions of Guangxi mandarin orange peel, Meizhou shatian pomelo peel, Sichuan lemon peel, and Jiangxi navel orange peel without UA-ATPE treatment, respectively; (E), (F), (G), and (H) represent the solutions of Guangxi citrus peel, Meizhou shatian pomelo peel, Sichuan lemon peel, and Jiangxi navel orange peel with UA-ATPE treatment, respectively.





Fig S2. A plot of the random distribution of test statistic Q^2^ values for the OPLS-DA permutation test.





Fig S3. Comparison of antioxidant potential composite (APC) indices of different citrus peel upper phase extracts. GX: upper phase extract of Guangxi mandarin orange peel; MZ: upper phase extract of Meizhou shatian pomelo peel; SC: upper phase extract of Sichuan lemon peel; JX: upper phase extract of Jiangxi Gannan navel orange peel.





Fig S4. Total ion chromatograms of upper phase extracts from four different citrus peels. GX: upper phase extract of Guangxi citru peel; MZ: upper phase extract of Meizhou shatian pomelo peel; SC: upper phase extract of Sichuan lemon peel; JX: upper phase extract of Jiangxi Gannan navel orange peel.

**Tables （S1-S9)**

Table S1. Volumes of upper and lower phases with UA-ATPE treatment of different citrus peels

| volume (mL) | GX | MZ | SC | JX |
| --- | --- | --- | --- | --- |
| upper phases | 59.90 ± 0.02 a | 49.00 ± 0.02 b | 59.20 ± 0.01 a | 51.20 ± 0.03 b |
| lower phases | 39.20 ± 0.02 b | 51.20 ± 0.02 a | 41.00 ± 0.01 b | 50.00 ± 0.03 a |

GX: upper phase extract of Guangxi mandarin orange peel; MZ: upper phase extract of Meizhou shatian pomelo peel; SC: upper phase extract of Sichuan lemon peel; JX: upper phase extract of Jiangxi Gannan navel orange peel. Means with different letters in the same row differ significantly (*p* < 0.05).

Table S2. Total flavonoid, total phenol, and protein extraction yields in four varieties of citrus peels

| Yield (mg/g fw) | Mandarin orange | Shatian pomelo | Lemon | Navel orange |
| --- | --- | --- | --- | --- |
| Total flavonoid | 9.13 ± 0.21 a | 4.07 ± 0.25 c | 5.45 ± 0.51 b | 3.31 ± 0.05 d |
| Total phenolic | 3.94 ± 0.03 b | 2.35± 0.03 c | 4.74± 0.06 a | 2.18± 0.02 d |
| Protein | 0.45 ± 0.02ab | 0.22 ± 0.01 c | 0.46 ± 0.03 a | 0.41 ± 0.02 b |

Fw: fresh citrus peels weight. Means with different letters in the same row differ significantly (*p* < 0.05).

Table S3. Contents of forty flavonoids determined in different UPEs

| Metabolite | GX (ng/mL) | MZ (ng/mL) | SC (ng/mL) | JX (ng/mL) |
| --- | --- | --- | --- | --- |
| Rutin | 2942.82 ± 401.28 a | 8.63 ± 1.42 c | 1513.8 ± 66.47 b | 251.71 ± 12.68 c |
| Daidzin | 0.5 ± 0.6 b | 0.57 ± 0.83 b | 5.63 ± 0.37 a | 1.22 ± 0.73 b |
| Genistin | 60.25 ± 8.82 b | 3.13 ± 0.09 c | 114.3 ± 11.84 a | 7.74 ± 0.75 c |
| p-Coumaric acid | 3404.9 ± 215.97 a | NA | NA | 337.77 ± 56.49 b |
| Luteolin | 2788.43 ± 289.92 a | 16.09 ± 1.16 b | 25.15 ± 6.83 b | 2507.5 ± 156.55 a |
| Quercetin 3-O-glucoside | 122.05 ± 11.45 a | 13.86 ± 1.59 c | 18.95 ± 3.15 c | 88.85 ± 5.79 b |
| Daidzein | 20.26 ± 5.57 a | 13.01 ± 2.47 a | 14.58 ± 8.27 a | 16.18 ± 6.96 a |
| Genistein | 2.53 ± 0.26 a | 1.4 ± 0.65 b | 1.51 ± 0.05 b | 1.54 ± 0.09 b |
| Eriodictyol | 27.03 ± 2.56 a | 1.41 ± 0.02 c | 21.5 ± 3.85 b | 22.47 ± 2.44 ab |
| Sakuranetin | 0.17 ± 0.05 a | 0.69 ± 0.63 a | 0.06 ± 0.03 a | 0.03 ± 0.02 a |
| Luteolin-7-O-glucoside | 419.15 ± 46.96 a | 99.01 ± 1.81 b | 374.58 ± 22.85 a | 122.82 ± 6.26 b |
| Puerarin | 1.77 ± 0.11 b | 1.91 ± 0.52 b | 0.75 ± 0.14 c | 2.71 ± 0.09 a |
| Phenylalanine | 1541.33 ± 114.97 c | 3195.36 ± 102.92 b | 607.57 ± 46.86 d | 3490.95 ± 212.05 a |
| Naringin | NA | 27116.54 ± 1625.84 | NA | NA |
| Glycetein | 0.66 ± 0.07 a | 0.83 ± 0.48 a | 0.54 ± 0.03 a | 0.62 ± 0.03 a |
| Formononetin | NA | 1.12 ± 0.34 | NA | NA |
| Isoliquiritigenin | NA | 2.14 ± 0.31 | NA | NA |
| Biochanin A | 1.57 ± 0.03 a | 1.68 ± 0.24 a | 1.25 ± 0.02 b | 1.67 ± 0.08 a |
| Naringenin | 35.74 ± 2.34 a | 4.91 ± 0.23 c | 2.63 ± 0.12 c | 20.43 ± 1.5 b |
| Epicatechin | 2.37 ± 0.21 c | 13.49 ± 1.82 b | 3.07 ± 0.27 c | 22.45 ± 2.04 a |
| Butin | NA | 1.09 ± 0.59 a | 0.78 ± 0.11 a | NA |
| Quercitrin | 1.00 ± 0.03 a | 0.82 ± 0.52 a | 0.74 ± 0.05 a | 0.75 ± 0.06 a |
| Liquiritigenin | NA | 0.56 ± 0.65 | NA | NA |
| Apigenin | 3.24 ± 0.08 a | 2.2 ± 0.32 b | 2.12 ± 0.02 b | 1.94 ± 0.01 b |
| (+)-Gallocatechin | NA | 784.13 ± 124.13 | NA | NA |
| Ferulic acid | 938.86 ± 45.6 a | 60.14 ± 4.55 d | 205.98 ± 20.68 c | 407.94 ± 50.16 b |
| Vitexin/Isovitexin | 89.07 ± 7.34 b | 36.04 ± 2.72 c | 316.59 ± 56.55 a | 30.52 ± 2.24 c |
| Kaempferide | NA | 0.99 ± 0.49 a | 0.66 ± 0.15 a | 0.36 ± 0.07 a |
| Hesperitin | 79880.1 ± 3801.69 b | 12681.88 ± 994.9 c | 224965.94 ± 34547.05 a | 26519.9 ± 4007.93 c |
| Tangeretin | 57434.62±1052.19 a | 15507.3 ± 957.59 c | 5068.85 ± 687.52 d | 24585.71 ± 1857.34 b |
| Kaempferol | NA | NA | NA | NA |
| Taxifolin | NA | NA | NA | NA |
| Myricetin | NA | NA | NA | NA |
| Dihydrokaempferol | NA | NA | NA | NA |
| Glycitin | NA | NA | NA | NA |
| Quercetin | NA | NA | NA | NA |
| Isorhamnetin | NA | NA | NA | NA |
| Catechin | NA | NA | NA | NA |
| (-)-Epigallocatechin | NA | NA | NA | NA |
| Chrysin | NA | NA | NA | NA |

GX: upper phase extract of Guangxi mandarin orange peel; MZ: upper phase extract of Meizhou shatian pomelo peel; SC: upper phase extract of Sichuan lemon peel; JX: upper phase extract of Jiangxi Gannan navel orange peel. Means with different letters in the same row differ significantly (*p* < 0.05).

Table S4. Contents of forty flavonoids determined in four citrus-peel varieties.

| Metabolite | Mandarin orang  （μg/100g fw） | Shatian pomelo  （μg/100g fw） | Lemon  （μg/100g fw） | Navel orange  （μg/100g fw） |
| --- | --- | --- | --- | --- |
| Rutin | 4406.87 ± 600.91 a | 10.58 ± 1.73 c | 2240.42 ± 98.38 b | 322.19 ± 16.24 c |
| Daidzin | 0.75 ± 0.91 b | 0.7 ± 1.01 b | 8.33 ± 0.54 a | 1.56 ± 0.94 b |
| Genistin | 90.23 ± 13.21 b | 3.84 ± 0.12 c | 169.17 ± 17.52 a | 9.91 ± 0.96 c |
| p-Coumaric acid | 5098.84 ± 323.41 a | NA | NA | 432.35 ± 72.3 b |
| Luteolin | 4175.67 ± 434.16 a | 19.71 ± 1.42 c | 37.22 ± 10.11 c | 3209.6 ± 200.38 b |
| Quercetin 3-O-glucoside | 182.77 ± 17.14 a | 16.98 ± 1.94 c | 28.04 ± 4.66 c | 113.73 ± 7.41 b |
| Daidzein | 30.33 ± 8.34 a | 15.93 ± 3.03 a | 21.57 ± 12.24 a | 20.7 ± 8.91 a |
| Genistein | 3.79 ± 0.39 a | 1.72 ± 0.8 b | 2.23 ± 0.07 b | 1.97 ± 0.11 b |
| Eriodictyol | 40.48 ± 3.84 a | 1.73 ± 0.02 c | 31.83 ± 5.7 b | 28.76 ± 3.12 b |
| Sakuranetin | 0.25 ± 0.08 a | 0.84 ± 0.77 a | 0.09 ± 0.05 a | 0.04 ± 0.02 a |
| Luteolin-7-O-glucoside | 627.68 ± 70.32 a | 121.29 ± 2.22 c | 554.38 ± 33.81 b | 157.21 ± 8.02 c |
| Puerarin | 2.66 ± 0.16 a | 2.34 ± 0.64 b | 1.1 ± 0.2 c | 3.47 ± 0.11 b |
| Phenylalanine | 2308.14 ± 172.17 c | 3914.32 ± 126.08 b | 899.21 ± 69.35 d | 4468.41 ± 271.43 a |
| Naringin | NA | 33217.77± 1991.66 | NA | NA |
| Glycetein | 0.98 ± 0.11 a | 1.02 ± 0.59 a | 0.81 ± 0.04 a | 0.79 ± 0.04 a |
| Formononetin | NA | 1.37 ± 0.42 | NA | NA |
| Isoliquiritigenin | NA | 2.62 ± 0.38 | NA | NA |
| Biochanin A | 2.35 ± 0.04 a | 2.05 ± 0.29 ab | 1.85 ± 0.02 c | 2.14 ± 0.1 ab |
| Naringenin | 53.52 ± 3.51 a | 6.02 ± 0.28 c | 3.89 ± 0.18 c | 26.15 ± 1.93 b |
| Epicatechin | 3.55 ± 0.31 c | 16.52 ± 2.23 b | 4.55 ± 0.4 c | 28.73 ± 2.61 a |
| Butin | NA | 1.34 ± 0.72 a | 1.15 ± 0.16 a | NA |
| Quercitrin | 1.5 ± 0.05 a | 1.01 ± 0.64 a | 1.1 ± 0.08 a | 0.96 ± 0.08 a |
| Liquiritigenin | NA | 0.68 ± 0.8 | NA | NA |
| Apigenin | 4.85 ± 0.12 a | 2.69 ± 0.4 c | 3.14 ± 0.03 b | 2.49 ± 0.02 c |
| (+)-Gallocatechin | NA | 960.56 ± 152.06 | NA | NA |
| Ferulic acid | 1405.95 ± 68.28 a | 73.67 ± 5.58 d | 304.85 ± 30.6 c | 522.17 ± 64.2 b |
| Vitexin/Isovitexin | 133.38 ± 10.99 b | 44.15 ± 3.33 c | 468.55 ± 83.7 a | 39.07 ± 2.87 c |
| Kaempferide | 0 | 1.22 ± 0.61 a | 0.98 ± 0.22 a | 0.47 ± 0.09 a |
| Hesperitin | 119620.44 ± 5693.04 b | 15535.31 ± 1218.75 c | 332949.58 ± 51129.63 a | 33945.48 ± 5130.15 c |
| Tangeretin | 86008.35± 1575.65 a | 18996.45± 1173.05 c | 7501.89 ± 1017.52 d | 31469.7± 2377.39 b |
| Kaempferol | NA | NA | NA | NA |
| Taxifolin | NA | NA | NA | NA |
| Myricetin | NA | NA | NA | NA |
| Dihydrokaempferol | NA | NA | NA | NA |
| Glycitin | NA | NA | NA | NA |
| Quercetin | NA | NA | NA | NA |
| Isorhamnetin | NA | NA | NA | NA |
| Catechin | NA | NA | NA | NA |
| (-)-Epigallocatechin | NA | NA | NA | NA |
| Chrysin | NA | NA | NA | NA |

Fw: fresh citrus peels weight. Means with different letters in the same row differ significantly (*p* < 0.05).

Table S5. Comparison of the total flavonoid, total phenolic and protein contents of UPEs from four citrus varieties

| Content (mg/mL) | GX | MZ | SC | JX |
| --- | --- | --- | --- | --- |
| TF | 0.61 ± 0.01 a | 0.33 ± 0.02 b | 0.37 ± 0.03 b | 0.26 ± 0.00 c |
| TP | 0.26 ± 0.00 b | 0.19 ± 0.00 c | 0.32 ± 0.01 a | 0.17 ± 0.00 d |
| Protein | 0.03 ± 0.01 a | 0.02 ± 0.02 b | 0.03 ± 0.03 a | 0.03 ± 0.00 a |

TF: total flavonoid content; TP: total phenolic content.

Table S6. The extraction yields of pectin from four different citrus peels

| Yields (%) | Citrus | Pomelo | Lemon | Orange |
| --- | --- | --- | --- | --- |
| UA-ATPE | 3.73 ± 0.02 d | 6.22 ± 0.02 b | 4.96 ± 0.03 c | 8.47 ± 0.03 a |
| Acid-extraction | 3.46 ± 0.03 a | 0.98 ± 0.01 d | 2.23 ± 0.02 c | 3.18 ± 0.02 b |

Table S7. Comparison of antioxidant activities of UPEs from selected citrus peels

| Methods | Concentration (v/v) | GX | MZ | SC | JX |
| --- | --- | --- | --- | --- | --- |
| ABTS | 1.3% | 23.58 ± 1.80 a | 16.03 ± 1.61 b | 9.62 ± 0.82 c | 14.30 ± 2.93 b |
|  | 2.5% | 42.75 ± 4.36 a | 27.59 ± 5.54 b | 26.79 ± 0.74 b | 32.00 ± 1.21 b |
|  | 5.0% | 74.55 ± 1.23 a | 67.00 ± 4.42 ab | 63.53 ± 2.72 b | 62.46 ± 2.98 b |
|  | 10.0% | 96.86 ± 1.77 a | 95.12 ± 0.62 a | 75.75 ± 1.23 b | 94.99 ± 1.98 a |
|  | 20.0% | 100.73 ± 0.09 a | 95.53 ± 0.09 b | 99.13 ± 0.41 a | 100.27 ± 0.19 a |
| ·OH | 1.3% | 24.76 ±2.35 c | 65.14 ± 0.78 b | 10.65 ± 1.22 d | 74.83 ± 1.69 a |
|  | 2.5% | 37.07 ± 3.13 c | 88.24 ± 1.22 a | 55.33 ± 1.41 b | 86.86 ± 0.34 a |
|  | 5.0% | 66.67 ±2.88 c | 96.41 ± 0.09 a | 69.16 ± 3.11 | 89.21 ± 0.20 b |
|  | 10.0% | 82.99 ± 7.53 b | 98.04 ± 0.09 a | 87.14 ± 1.37 b | 91.42 ± 5.32 a |
|  | 20.0% | 85.2 ± 0.71 b | 101.02 ± 0.36 a | 87.69 ± 0.68 b | 104.56 ± 3.58 a |
| O_2_^·-^ | 1.3% | 4.17 ± 0.29 a | 4.03 ± 0.12 a | 0.51 ± 0.44 b | 5.43 ± 1.03 a |
|  | 2.5% | 22.53 ± 0.19 a | 13.4 ± 0.75 b | 9.06 ± 0.91 c | 20.37 ± 1.32 a |
|  | 5.0% | 38.43 ± 0.78 a | 38.3 ± 1.51 a | 25.13 ± 0.98 b | 27.15 ± 0.22 b |
|  | 10.0% | 64.13 ± 1.26 a | 61.22 ± 0.91 a | 48.44 ± 1.01 b | 62.18 ± 1.64 a |
|  | 20.0% | 82.2 ± 1.56 a | 81.46 ± 1.36 a | 71.07 ± 0.99 b | 83.03 ± 1.35 a |

**Table S8. Effects of UPEs from selected citrus peels on HaCaT cell viability**

| Concentration (%) | GX | MZ | SC | JX |
| --- | --- | --- | --- | --- |
| 0.06% | 132.64 ± 5.11 a | 139.40 ± 3.40 a | 117.31 ± 3.67 b | 139.67 ± 10.18 a |
| 0.25% | 128.49 ± 6.88 a | 123.57 ± 9.14 a | 111.72 ± 8.38 b | 120.11 ± 8.76 a |
| 1.00% | 112.91 ± 8.64 b | 124.49 ± 5.91 a | 117.85 ± 3.11 b | 101.31 ± 4.43 c |

**Table S9. Effects of UPEts from selected citrus peels on BJ cell viability**

| Concentration (%) | GX | MZ | SC | JX |
| --- | --- | --- | --- | --- |
| 0.06% | 92.64 ± 3.53 b | 95.95 ± 6.57 b | 99.25 ± 0.17 ab | 107.7 ± 1.01 a |
| 0.25% | 95.46 ± 2.06 a | 99.17 ± 4.02 a | 95.74 ± 8.83 a | 100.41 ± 5.66 a |
| 1.00% | 81.43 ± 2.73 b | 99.59 ± 2.55 a | 84.87 ± 5.35 b | 96.29 ± 0.85 a |
